# Liquid Foam Templates Associated with the Sol-Gel Process for Production of Zirconia Ceramic Foams

**DOI:** 10.3390/ma6051967

**Published:** 2013-05-10

**Authors:** Cristiane Carolina Beozzo, Marinalva Aparecida Alves-Rosa, Sandra Helena Pulcinelli, Celso Valentim Santilli

**Affiliations:** Institute of Chemistry, Universidade Estadual Paulista—UNESP, P.O. BOX 355, Araraquara 14800-900, Brazil; E-Mails: cris.beozzo@gmail.com (C.C.B.); sandrap@iq.unesp.br (S.H.P.); santilli@iq.unesp.br (C.V.S.)

**Keywords:** ceramic foam, liquid-foam templates, sol-gel, surfactant, thermal treatment

## Abstract

The unique properties of ceramic foams enable their use in a variety of applications. This work investigated the effects of different parameters on the production of zirconia ceramic foam using the sol-gel process associated with liquid foam templates. Evaluation was made of the influence of the thermal treatment temperature on the porous and crystalline characteristics of foams manufactured using different amounts of sodium dodecylsulfate (SDS) surfactant. A maximum pore volume, with high porosity (94%) and a bimodal pore size distribution, was observed for the ceramic foam produced with 10% SDS. Macropores, with an average size of around 30 μm, were obtained irrespective of the SDS amount, while the average size of the supermesopores increased systematically as the SDS amount was increased up to 10%, after which it decreased. X-ray diffraction analyses showed that the sample treated at 500 °C was amorphous, while crystallization into a tetragonal metastable phase occurred at 600 °C due to the presence of sulfate groups in the zirconia structure. At 800 and 1000 °C the monoclinic phase was observed, which is thermodynamically stable at these temperatures.

## 1. Introduction

The useful properties of porous ceramics have increased interest in the development of new routes for producing materials with tailored porous structures. Ceramic foams belong to an emerging class of porous materials composed of a continuous solid phase that provides the scaffold for a large volume fraction of gas-filled pores [1]. The ceramic phase composition and the porous structure must be carefully selected to meet the needs of any particular application. Recent developments include the use of porous ceramics as catalyst supports [2], in phase separation processes [3], in biomedical materials [4], and in chromatography columns [5], amongst other uses.

The process employed to generate the porosity is critical since the porous structure determines the potential applications of the material. Hard templates based on polymers or natural sponges are common in industry [6,7]. This method, known as replication, is widely used to obtain reticulated foams possessing high porosity and permeability, and is especially useful in bone scaffolds for stimulating the natural regeneration of bone [7]. Although this is a straightforward procedure for the manufacture of porous materials, careful attention must be paid to the step involving infiltration of the ceramic slurry, because blockage of the scaffolds during this step could cause inner defects in the ceramic foam [4].

Fine control of pore structure has been achieved with methods that employ the direct foaming process [6,8]. The main techniques reported in the literature include the use of gelcasting [9], preceramic polymers [10], spinodal decomposition (phase separation) [5], and emulsions and liquid foams associated with the sol-gel process [8,11]. The sol-gel process is essential for control of the porous microstructure in the two latter cases. Compared to other methods that involve the use of polymers for guiding or shaping the pores, the sol-gel process releases smaller amounts of environmentally harmful substances during template elimination. Spinodal decomposition requires strict control of process parameters [5,12], while soft templates, such as emulsions and liquid foams, represent simple and direct methods for tailoring properties associated with the porous structure [8]. The use of the sol-gel process, integrated with soft templates, enables gelation of the liquid template, preventing collapse of the incipient porous structure during the drying and thermal treatment steps [13,14]. 

Emulsion pore templates use an immiscible phase to produce droplets inside the sol-gel solution containing the ceramic precursors. Materials possessing controlled microstructures that have been developed using emulsion templates include silica, alumina, titania, and zirconia foams with porosities of around 90% and a variety of pore sizes [11,13,15,16]. In order to create bubbles in the sol-gel solution, liquid foams require a gas that may be either generated [17] or introduced into the liquid system [8]. After gelation and drying, pores are formed at the locations of the bubbles in the liquid phase [8,18]. Distinct porous structures can be obtained using emulsion templates, but the presence of a liquid phase (such as oil) that needs to be removed to create the pores is a disadvantage of this route. Cracks can appear during oil evaporation due to the high steam pressure of this phase during heating [13].

In the present work, the sol-gel process associated with liquid foams as templates of pores was used to produce zirconia ceramic foams. This is a further development of the work described in the recent paper concerning the direct foaming method based on an emulsion template [13]. The use of the basic zirconium sulfate (BZS) aqueous suspension developed by Chiavacci *et al.* [19] allows control of the initiation of gelation; the ceramic phase microstructure can be adjusted by the sol-gel transition, which depends on the composition of the suspension. The composition of the ceramic phase determines the potential applications of the final material as catalyst supports and thermal insulation. Since the porous properties are influenced by the stability of the liquid foam, sodium dodecylsulfate (SDS) was used to stabilize the air-liquid interface [18]. The effects of the SDS amount and the thermal treatment temperature on the porous microstructure were analyzed, because both the porosity and the crystalline phase of the zirconia are extremely important for its use as a catalyst support. An advantage of the system is the composition of the ceramic phase, which presents acidic active sites when sulfate groups are retained, allowing the material to be used in catalytic dehydration reactions, while in the absence of these sulfate groups the presence of adsorbed metals on the zirconia surface enables application in other catalytic reactions.

## 2. Experimental Section 

### 2.1. Zirconia Foams Preparation

The basic zirconium sulfate aqueous sol was prepared according to the procedure described by Chiavacci *et al.* [19]. This consisted of drop-by-drop addition of a 1.5 mol L^−1^ zirconium oxychloride solution (Sigma-Aldrich, 98%) to a 1.5 mol L^−1^ sulfuric acid solution (Mallinckrodt, 96%) at 80 °C until a ZrOCl_2_:H_2_SO_4_ ratio of 15:1 was reached. The resultant clear suspension was cooled to room temperature and dialyzed against bi-distilled water at a V_sol_:V_water_ ratio of 1:10 for 24 h. The zirconium concentration was increased from 0.5 to 3.5 mol L^−1^ by solvent evaporation at 55 °C using a rotary evaporator.

**Figure 1:**
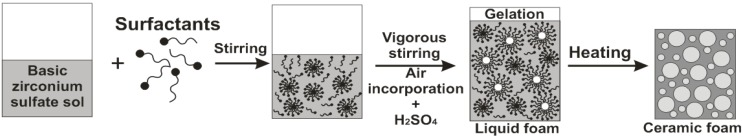
Schematic representation of the preparation of zirconia ceramic foams by the sol-gel process associated with a liquid foam template.

The liquid foam was prepared by adding different amounts of the SDS surfactant to the aqueous sol, followed by vigorous stirring. A schematic representation of the preparation process is shown in Figure 1, which also indicates the ability to create a bimodal pore size distribution when some of the surfactant micelles are swollen by the incorporation of air (large pores), while others remain unswollen (small pores). The amounts of SDS used were 5%, 7%, 10%, 15%, and 20%, calculated using the relationship %SDS = (mass_SDS_/mass_total_) × 100%, corresponding to SDS molar compositions of 3.4 × 10^−4^, 4.9 × 10^−4^, 7.2 × 10^−4^, 1.4 × 10^−3^, and 1.6 × 10^−3^, respectively. Aeration was promoted by vigorous stirring for 2 min, using a mechanical stirrer operated at 2000 rpm. After foaming of the liquid, gelation was induced by adding sulfuric acid to this system until a Zr/SO_4_ molar ratio of 3:1 was reached. The resulting gel was aged inside closed vessels at 25 °C for 9 days. The wet gel was first dried for 48 h at room temperature and then in a conventional oven at 55 °C for 48 h. Calcination of the foam was carried out in a conventional muffle furnace by increasing the temperature in successive steps: 2 h at 90 °C, 2 h at 350 °C, and 3 h at 600 °C, in order to eliminate the remaining water and the residual carbonaceous material derived from the surfactant. One sample was treated without SDS, as a control, and is referred to here as unfoamed (or 0% SDS). This sample received the same treatment as the others.

### 2.2. Zirconia Foams Characterization

The porous structure was characterized using skeletal (*ρ*_s_) and bulk (*ρ*_b_) density measurements employing helium (Accupyc 1330, Micromeritics) and dry-fluid (GeoPyc 1360, Micromeritics) pycnometry, respectively. The open porosity (*P*) was calculated by the relation *P* = (1 − *ρ*_b_/*ρ*_s_). The pore size distribution was determined by mercury porosimetry using an Autopore III (Micromeritics) instrument, and the pore diameter was calculated according to the Washburn equation [20]. Measurements of nitrogen adsorption-desorption were conducted in order to determine the specific surface area and the presence of mesopores (2 nm < *d* < 50 nm). The surface area was estimated by the BET equation [21]. The analyses were performed at liquid nitrogen temperature and a relative pressure interval of between 0.002 and 0.998, using an ASAP 2010 instrument (Micromeritics). The samples were evacuated for 14 h at 100 °C under 10 μPa of vacuum, and the type and size of pores was examined by scanning electron microscopy (SEM), using a Philips XL 30 instrument. The samples were deposited onto aluminum sample holders with conductive silver ink, and sputtered with gold.

Thermogravimetric analyses were performed using a TA Instruments SDT Q600 Simultaneous DTA/TG instrument in order to determine the thermal treatment temperatures and analyze the events that occurred during heating, such as the elimination of organic compounds and sulfate species. The samples were heated at 10 °C min^−1^ under a flow of oxygen (100 cm^3^ min^−1^) and the thermal events were monitored between 25 and 1000 °C.

The zirconia crystalline phases present in the ceramic foams that had been thermally treated at different temperatures were analyzed by X-ray powder diffraction (XRPD) using a Siemens D5000 diffractometer, with CuK_α_ radiation and a step of 0.03° (2θ). Phase identification was achieved using the X’Pert High Score program and the crystallographic pattern files for the tetragonal phase (PDF-79-1771) and the monoclinic phase (PDF-83-0943).

## 3. Results and Discussion

### 3.1. Effect of Sodium Dodecylsulfate (SDS) Amount

Surfactants have an important function in the preparation of foams due to their ability to stabilize the air-liquid interface in the liquid foam templates, and consequently define the porous structure in the final ceramic material. Table 1 shows the porous characteristics of zirconia foams prepared with different amounts of SDS, which preserved the monolithic format up to an SDS concentration of 10%. The data were obtained by helium and dry-fluid pycnometry and Hg porosimetry. A maximum specific pore volume (3.19 cm^3^ g^−1^) was obtained for 10% SDS. It was evident that an excess of SDS could be harmful to the stability of the liquid foam, as observed for the sample prepared with 20% SDS, where there was coalescence of pores or cracking in pore walls during removal of the template. As a result, a minimum pore volume (0.80 cm^3^ g^−1^) was observed for this sample, which was close to the value observed for the unfoamed sample (0% SDS). High porosity (~93%) and low bulk density (~0.3 g cm^−3^) were observed for samples prepared using SDS amounts of between 5% and 15%. The small differences in porosity and density could have been due to the processes of template removal. Similar experiments using silica and SDS/CTAB (cetyl trimethyl ammonium bromide) mixtures have shown an increase in porosity and consequent decrease in bulk density with increasing amounts of SDS, reaching a maximum open porosity value of about 80% [22]. Porosities similar to or higher than those observed here are only observed for aerogels, which require high pressure and temperature for supercritical drying [23].

**Table 1:** Porous characteristics of zirconia foams prepared with different amounts of sodium dodecylsulfate (SDS).

SDS amount (wt %)	Specific pore volume (cm^3^ g^−1^)	Bulk density (g cm^−3^)	Porosity (%)	Mean macropore size (μm)	Mean supermesopore size (μm)
0	0.70 ± 0.02	1.628 ± 0.007	64.5 ± 0.3	–	1.83 ± 0.03
5	3.01 ± 0.03	0.278 ± 0.002	93.7 ± 0.6	17.5 ± 0.1	0.42 ± 0.01
7	2.82 ± 0.03	0.330 ± 0.003	92.8 ± 0.8	29.5 ± 0.6	0.96 ± 0.02
10	3.19 ± 0.05	0.292 ± 0.004	94 ± 1	13.6 ± 0.1	1.3 ± 0.1
15	2.70 ± 0.03	0.338 ± 0.004	91 ± 1	22.3 ± 0.2	0.92 ± 0.07
20	0.80 ± 0.06	0.80 ± 0.03	81 ± 3	94 ± 7	0.68 ± 0.06

**Figure 2:**
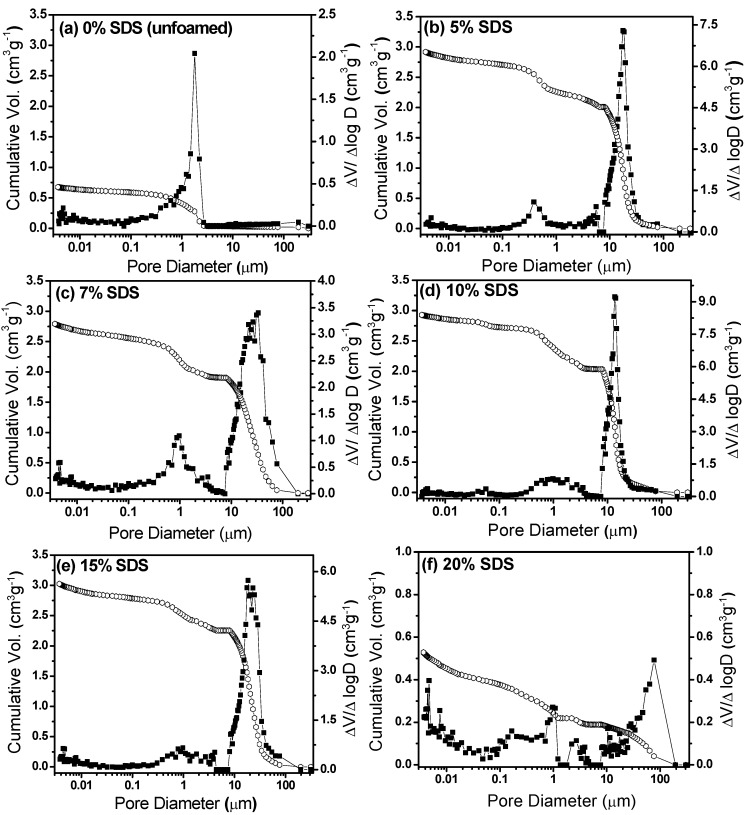
Cumulative and differential pore size distributions of ceramic foams prepared from liquid foams containing different amounts of SDS: (**a**) 0%; (**b**) 5%; (**c**) 7%; (**d**) 10%; (**e**) 15%; and (**f**) 20%.

The two last columns of Table 1 present the mean pore size values calculated from the cumulative and differential pore size distributions shown in Figure 2. The unfoamed sample only presented a few supermesopores (with a mean size of 1.83 μm) and had a specific volume of around 0.70 cm^3^ g^−1^ (Figure 2a). The foamed samples (Figure 2b,f) presented one main family of macropores (*d* > 4 μm) with high pore volume, together with a secondary family of supermesopores (*d* < 4 μm) [14]. The macropores were formed by air bubbles from the liquid foam templates, while the supermesopores resulted from the presence of an excess of SDS molecules in the system. This porous structure is promoted during the elimination of solvent from the gel network, when small slit cavities resulting from the non-parallel stacking of platelets can be formed [13,24]. While the macropores maintained diameters of around 20 μm and volumes in the region of 2.0 cm^3^ g^−1^ (Figure 2), the mean diameter and specific volume of the supermesopores increased from 0.42 μm and 0.51 cm^3^ g^−1^ to 1.31 μm and 0.71 cm^3^ g^−1^ for 5% and 10% SDS, respectively. Both the diameter and the volume decreased for SDS amounts higher than 10%. This behavior indicated that a certain fixed quantity of SDS molecules stabilized the air-liquid interface, limiting the quantity of air bubbles incorporated into the system during foaming. 

**Figure 3:**
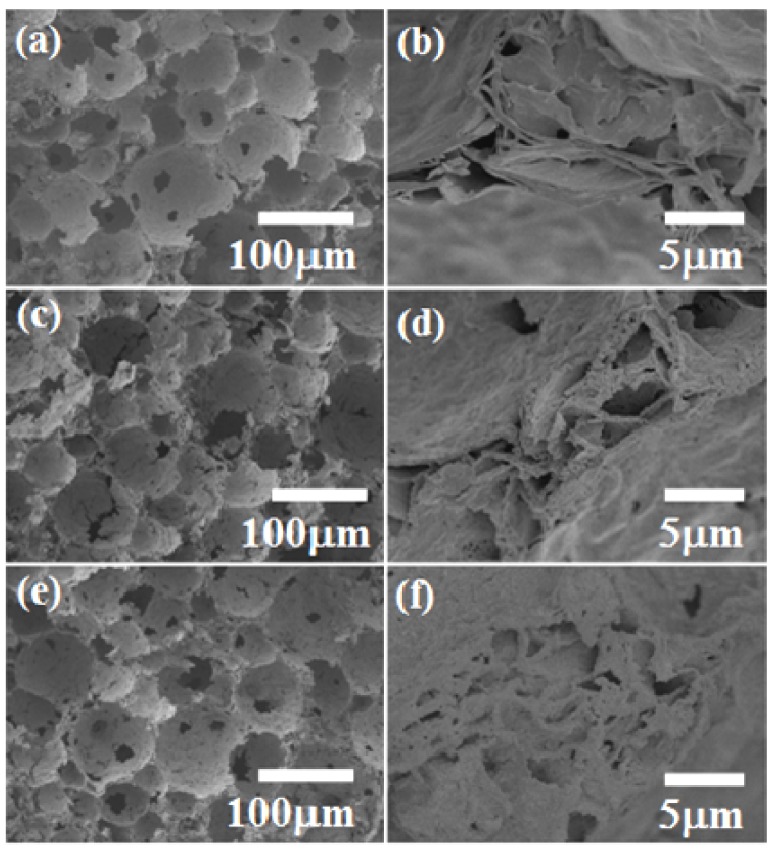
Scanning electron micrographs of zirconia ceramic foams prepared from liquid foams containing different amounts of SDS: (**a**,**b**) 5%; (**c**,**d**) 10%; (**e**,**f**) 15%.

Representative scanning electron microscopy (SEM) images of the ceramic foams (Figure 3) illustrate the effect of the SDS amount on the porous morphology of the samples calcined at 600 °C. Spherical macropores can be seen in the micrographs of Figure 3a,c,e, corresponding to the use of 5%, 10%, and 15% SDS, respectively. Higher magnification images of the structures present between the macropores (Figure 3b,d,f) reveal the supermesopores detected by Hg porosimetry. This confirms that the general structures of the foams consisted of spherical macropores surrounded by supermesopores, which were in fact inside the walls of the macropores. The main effect of the SDS amount was on the structures that composed the macropore walls, which were densest for the samples prepared with 5% SDS and had a more textured appearance when 10% and 15% SDS were used.

**Figure 4:**
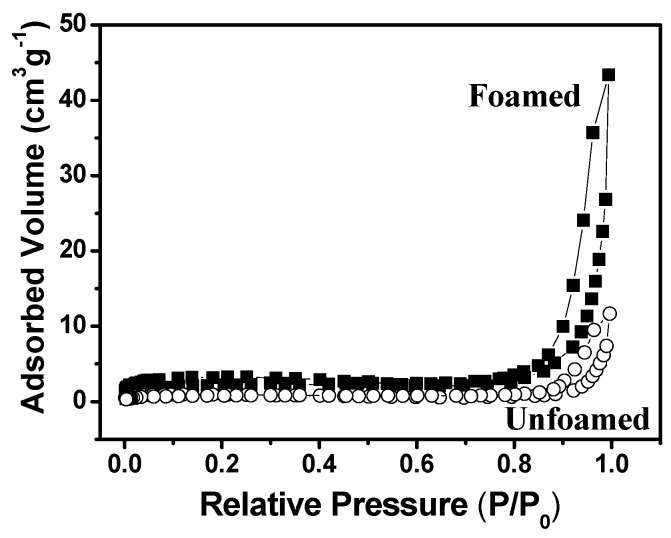
Nitrogen adsorption-desorption isotherms for foamed and unfoamed zirconia ceramics.

Nitrogen adsorption-desorption isotherm analysis was used to evaluate the samples in terms of surface area and the mesopore size distribution (2 nm < *d* < 50 nm). Figure 4 shows the N_2_ isotherms obtained for a representative foamed sample (7% SDS) and the unfoamed material. Both curves display a near vertical loop, characteristic of type H1 hysteresis and indicative of elongated (cylindrical) pores. The absence of a plateau at relative pressure near to 1 is typical of type II isotherms, frequently observed for macroporous solids with pore sizes exceeding 50 nm, reflecting the coexistence of both mesopores and macropores [16,21]. This is consistent with the presence of the two pore families revealed by Hg porosimetry (Figure 2). Nevertheless, the low volume of nitrogen adsorbed by the samples corresponded to small BET surface areas of around 7 m^2^ g^−1^. Small BET surface areas have also been reported for TiO_2_ foams prepared with air-liquid foam templates and SDS as surfactant [18].

### 3.2. Effect of Thermal Treatment Temperature

**Figure 5:**
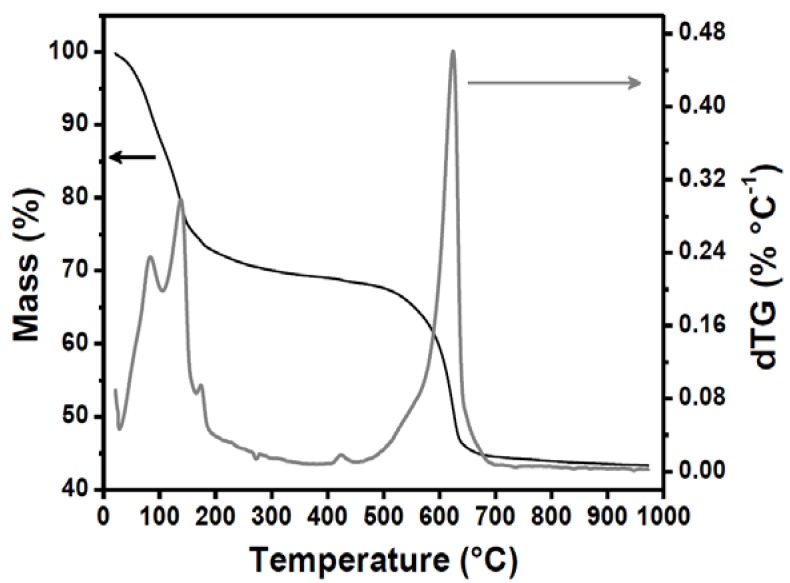
Thermogravimetric curves (TG and dTG) for the wet gel prepared using 10% SDS.

A crucial step in foam preparation is the thermal treatment process, which can improve or damage formation of the porous structure during the removal of volatile components. The influence of the thermal treatment temperature was evaluated by observing the porous microstructure of the sample prepared with 10% SDS, which presented the highest porosity. Thermal analysis of the wet gel permitted identification of temperatures that could damage the porous structure. The thermogravimetric curve (Figure 5) showed the existence of two distinct processes. The first started at room temperature and continued up to about 140 °C, with the loss of around 25% of the mass, and was attributed to the elimination of surface water. The second process, which occurred between around 500 and 650 °C, corresponded to a 23% mass loss and was related to the removal of carbon and sulfates, indicating that large quantities of these components had been grafted onto the foamed sample.

According to the literature [25,26], carbonaceous residues from the surfactant added to promote foaming may be eliminated from 400 °C, and in higher amounts above 500 °C, while sulfate decomposition occurs above 550 °C and is followed by the evolution of SO_3_ and/or SO_2_/SO, together with O_2_ and O. Since sulfated zirconia retains a fraction of the sulfate groups in the structure when heated at 600 °C, it presents the high surface acidity necessary for catalytic dehydration processes [27,28]. After removal of the sulfate groups above 700 °C, while maintaining the porous structure, zirconia foam can be impregnated with metal atoms for use in a variety of other catalytic processes [28].

**Figure 6:**
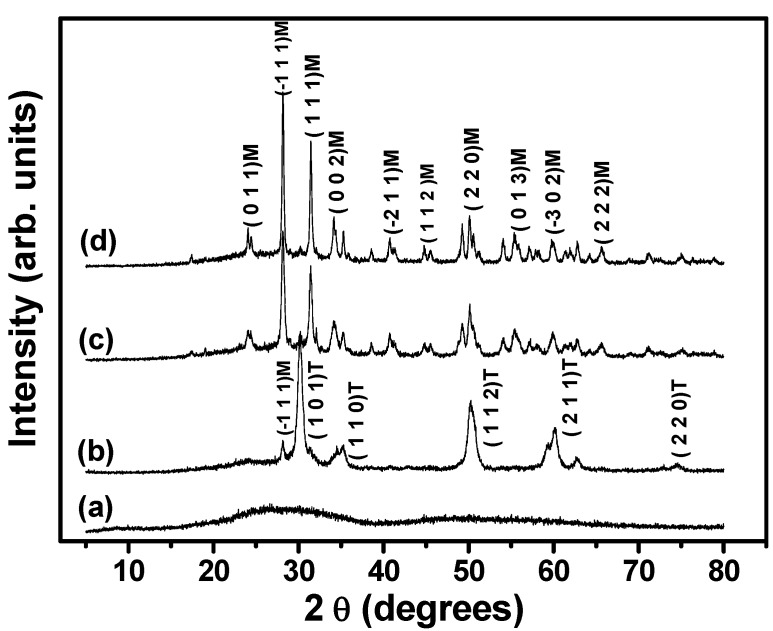
XRD patterns of zirconia foams fired at different temperatures: (**a**) 500 °C; (**b**) 600 °C; (**c**) 800 °C; (**d**) 1000 °C.

The temperatures that could influence both the porous and crystalline structures of the zirconia foams were obtained from the thermogravimetric measurements (Figure 5), considering the second phase of mass loss in which the surfactant and the sulfate groups were eliminated. Monolithic zirconia presents virtually no shrinkage during thermal treatment up to 500 °C, while TiO_2_ foam samples treated at the same temperature show 50% shrinkage [18]. Figure 6 presents the XRPD patterns obtained for samples heat-treated at 500, 600, 800, and 1000 °C. An amorphous structure was observed for the sample heated at 500 °C, as evidenced by the absence of diffraction peaks. The tetragonal phase (PDF-79-1771), evidenced by the peaks centered at about 30° and 50°, was predominant for the sample heated at 600 °C. The occurrence of the metastable tetragonal phase was possibly due to surface stabilization effects caused by the grafting of the sulfate [27]. For the samples heated at 800 and 1000 °C, only the monoclinic crystalline phase was identified (PDF-83-0943), since all the sulfate groups had been eliminated.

**Figure 7:**
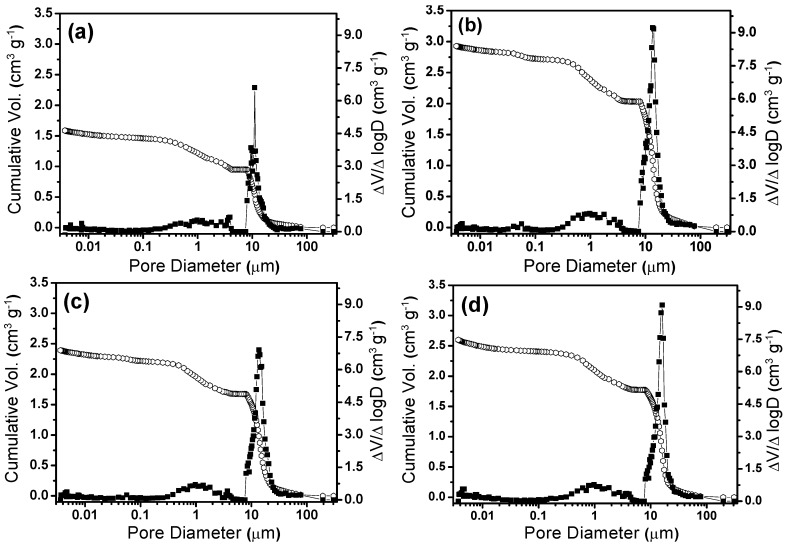
Cumulative and differential pore size distributions for ceramic foams fired at different temperatures: (**a**) 500 °C; (**b**) 600 °C; (**c**) 800 °C; (**d**) 1000 °C.

The values obtained for the specific pore volume, bulk density, porosity, and average pore size of the foams fired at different temperatures are presented in Table 2. The pore volume increased from 2.52 to 3.19 cm^3^ g^−1^ when the calcination temperature was increased from 500 to 600 °C, due to the removal of surfactant from the structure. A small decrease in the pore volume occurred when the temperature was increased from 800 to 1000 °C, probably due to a sintering densification process. The porosity and bulk density values were in good agreement with the pore volume, with values of around 93% and 0.3 g cm^−3^ achieved for the sample heated at 600 °C. The average macropore and supermesopore sizes shown in Table 2 were obtained from the differential pore size distribution shown in Figure 7. The macropore size showed a small increase, from about 11 to 15 μm, when the temperature was increased from 500 to 1000 °C, while a decrease of supermesopore size, from 2.3 to 1.15 μm, occurred over the same temperature interval. This effect can be seen in Figure 7, which shows the increase of macropore volume from 500 to 600 °C, together with the narrowing of the supermesopore size distribution as the temperature was increased. This effect could be explained by the closing of small cavities that had been formed around the pores by the surfactant. 

**Table 2:** Porous characteristics of zirconia foams prepared with 10% SDS and fired at different temperatures.

Thermal treatment	Total pore volume (cm^3^ g^−1^)	Bulk density (g cm^−3^)	Porosity (%)	Mean macropore size (µm)	Mean supermesopores size (µm)
500 °C	2.52 ± 0.01	0.358 ± 0.001	90.1 ± 0.3	10.9 ± 0.2	2.3 ± 0.2
600 °C	3.19 ± 0.05	0.293 ± 0.004	94 ± 1	13.6 ± 0.1	1.3 ± 0.1
800 °C	2.71 ± 0.05	0.331 ± 0.006	93 ± 2	14.0 ± 0.1	1.25 ± 0.07
1000 °C	2.6 ± 0.1	0.35 ± 0.02	91 ± 4	15.4 ± 0.1	1.15 ± 0.08

**Figure 8:**
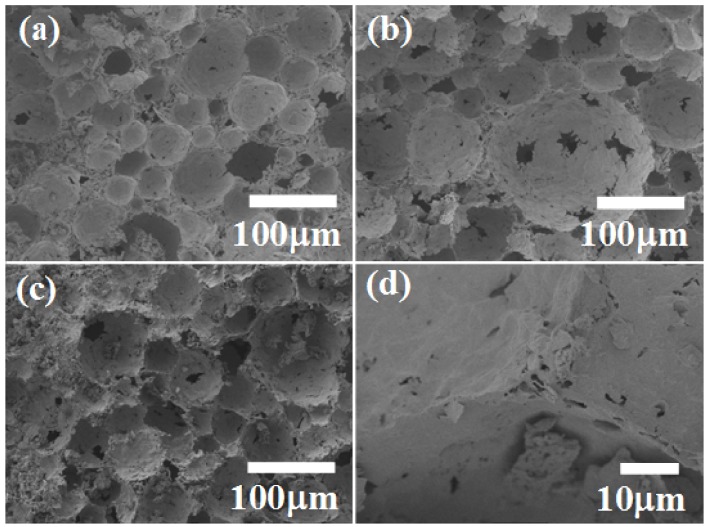
Scanning electron micrographs of zirconia ceramic foams fired at different temperatures: (**a**) 500 °C; (**b**) 800 °C; (**c**) and (**d**) 1000 °C.

Figure 8 shows micrographs of the ceramic foams calcined at different temperatures. Images of the sample fired at 600 °C are shown in Figure 3 c,d. At 500 °C (Figure 8a), the presence of closed pores is in agreement with the smaller pore volume observed by porosimetry, because mercury is unable to penetrate closed pores. The thermal analysis revealed that large quantities of compounds were eliminated between 500 and 700 °C, leading to the open-pore structure (Figure 3c,d and Figure 8b). Nevertheless, at 1000 °C the pore walls seemed to be denser than for the other samples, as evidenced in the magnified image of the junction between three closed pores (Figure 8c,d). This behavior is in agreement with the decreased pore volume at higher temperatures. As observed by Ahmed *et al.* [27], in addition to determining the structural and textural properties of sulfated zirconia, the calcination temperature affects the surface chemical properties, such as acidity, which govern the catalytic activity. The final porous and structural properties of sulfated zirconia ceramic foams can therefore be adjusted by control of the preparation parameters, especially the thermal treatment temperature and the type and amount of surfactant.

## 4. Conclusions 

The sol-gel process associated with liquid foam templates provides a simple and versatile route for the preparation of ceramic foams with high porosity and bimodal pore size distribution. The use of different amounts of SDS surfactant enables the shaping and stabilization of the porous structure, leading to the formation of monolithic zirconia foams containing spherical pores. The highest porosity (94%) was achieved for a sample prepared with 10% SDS, suggesting the existence of a practical limit to the amount of surfactant employed to stabilize the liquid foam prior to gelation. Low bulk densities (0.28 g cm^−3^) were observed due to the high pore volume. The values achieved for the porous properties have only been observed previously for aerogel-like materials produced under supercritical drying conditions. 

The thermal treatment temperature was a crucial determinant of the structural and textural properties of the foams. The sulfate species stabilized the tetragonal phase at 600 °C, but the removal of these species between 550 and 700 °C enabled formation of the monoclinic phase after calcination at 800 and 1000 °C.

The advantage of this foaming method is the ease with which the preparation parameters can be adjusted in order to alter characteristics of the materials including the porosity, pore size distribution, and crystalline structure. The surface properties and mass transport capacity of the materials enable their use in different catalytic applications.
